# Interactions Between the Serotonergic and Other Neurotransmitter Systems in the Basal Ganglia: Role in Parkinson’s Disease and Adverse Effects of L-DOPA

**DOI:** 10.3389/fnana.2020.00026

**Published:** 2020-06-04

**Authors:** Ana Muñoz, Andrea Lopez-Lopez, Carmen M. Labandeira, Jose L. Labandeira-Garcia

**Affiliations:** ^1^Laboratory of Cellular and Molecular Neurobiology of Parkinson’s Disease, Research Center for Molecular Medicine and Chronic Diseases (CIMUS), Deptartment of Morphological Sciences, Health Research Institute of Santiago de Compostela (IDIS), University of Santiago de Compostela, Santiago de Compostela, Spain; ^2^Networking Research Center on Neurodegenerative Diseases (CiberNed), Madrid, Spain; ^3^Department of Clinical Neurology, Hospital Alvaro Cunqueiro, University Hospital Complex, Vigo, Spain

**Keywords:** dopamine, dyskinesia, glutamate, Levodopa, Parkinson, serotonin, striatum

## Abstract

Parkinson’s disease (PD) is characterized by the progressive loss of dopaminergic neurons in the substantia nigra. However, other non-dopaminergic neuronal systems such as the serotonergic system are also involved. Serotonergic dysfunction is associated with non-motor symptoms and complications, including anxiety, depression, dementia, and sleep disturbances. This pathology reduces patient quality of life. Interaction between the serotonergic and other neurotransmitters systems such as dopamine, noradrenaline, glutamate, and GABA controls the activity of striatal neurons and are particularly interesting for understanding the pathophysiology of PD. Moreover, serotonergic dysfunction also causes motor symptoms. Interestingly, serotonergic neurons play an important role in the effects of L-DOPA in advanced PD stages. Serotonergic terminals can convert L-DOPA to dopamine, which mediates dopamine release as a “false” transmitter. The lack of any autoregulatory feedback control in serotonergic neurons to regulate L-DOPA-derived dopamine release contributes to the appearance of L-DOPA-induced dyskinesia (LID). This mechanism may also be involved in the development of graft-induced dyskinesias (GID), possibly due to the inclusion of serotonin neurons in the grafted tissue. Consistent with this, the administration of serotonergic agonists suppressed LID. In this review article, we summarize the interactions between the serotonergic and other systems. We also discuss the role of the serotonergic system in LID and if therapeutic approaches specifically targeting this system may constitute an effective strategy in PD.

## Introduction

Parkinson’s disease (PD) is one of the most common neurodegenerative disorders, which is characterized by the progressive loss of dopaminergic neurons in the substantia nigra compacta (SNc). Dopamine replacement therapy using the precursor L-DOPA is the main treatment for the disease. However, long-term use of L-DOPA leads to the development of dyskinesias and non-motor manifestations (Espay et al., 2018), showing that the pathological process extends beyond the dopaminergic system and that other neurotransmitter systems such as the serotonergic system are involved.

The dorsal raphe nucleus (DRN) contains the largest group of serotonin-producing neurons, and changes in DRN function have been implicated in neuropsychiatric diseases and movement disorders (Hornung, 2010; Huot et al., 2011). Classical studies using tracing techniques and recent works using single-cell RNA sequencing, *in situ* hybridization and adeno-associated viruses technology showed a dense serotonergic innervation of basal ganglia, including the caudate nucleus and SNc (Dahlström and Fuxe, 1964; Lavoie and Parent, 1990; Muzerelle et al., 2016; Huang et al., 2019).

Nowadays, seven classes of serotonin receptors (5-HT_1–7_) and at least 15 receptor subtypes have been identified (Hoyer et al., 2002; Hannon and Hoyer, 2008). Some of these receptors (5-HT_2C_, 5-HT_6_, 5-HT_7_) may have a constitutive activity, which may be associated with pathophysiological conditions (De Deurwaerdère et al., 2020). Type 1A/1B and 2A receptors (5-HT_1A/1B_ and 5-HT_2A_) appear particularly interesting for PD (Huot and Fox, 2013).

## Interactions Between Serotonin and Other Neurotransmitters in the Basal Ganglia

**Figure 1:**
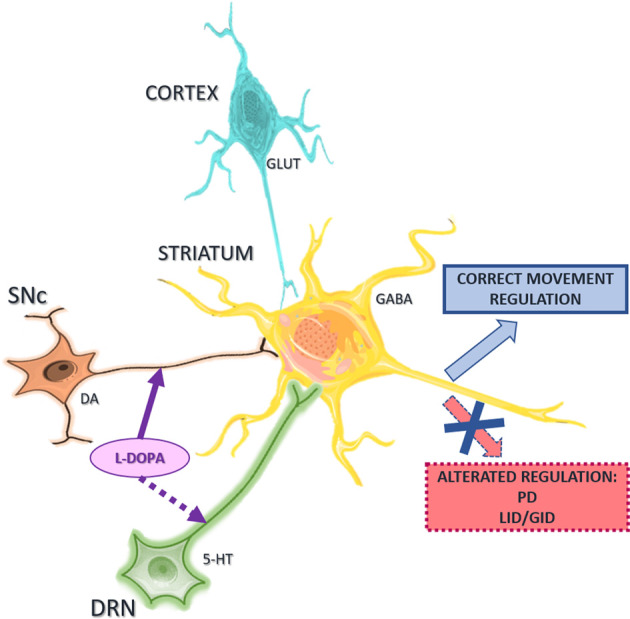
Schematic representation of the major neuronal neurotransmitter systems acting on striatal projection neurons. Interactions between serotonergic, glutamatergic, and dopaminergic systems control the activity of striatal neurons for correct regulation of movement. Abnormal interactions lead to abnormal movement and neurological disorders such as Parkinson’s disease (PD), L-DOPA-induced dyskinesia (LID) or graft-induced dyskinesia (GID). Abbreviations: SNc, substantia nigra pars compacta; DRN, dorsal raphe nucleus; 5-HT, serotonin; DA, dopamine, GLUT, glutamate. The figure was produced using Servier Medical Art (www.servier.com).

Several studies have highlighted a crucial role for the interactions between serotonergic and other neurotransmitter systems in movement control and pathophysiology of the basal ganglia (Di Matteo et al., 2008; Parent et al., 2011), and particularly PD (Ciranna, 2006; Figure 1).

### Interactions With the Dopaminergic System

Interactions between serotonin and dopamine have been investigated for decades, but the role of the serotonergic transmission in modulating the activity of dopaminergic neurons is still unclear (De Deurwaerdère and Di Giovanni, 2017; Ogawa and Watabe-Uchida, 2018). Several studies have suggested that serotonin input is inhibitory (Sinton and Fallon, 1988; Arborelius et al., 1993), as chronic serotonin transporter (SERT) blockade using serotonin-selective reuptake inhibitors (SSRIs) reduces dopaminergic signaling and elicits basal ganglia dysfunction (Morelli et al., 2011). However, DNR lesions did not affect SNc activity in other experiments (Kelland et al., 1990), and the lack of serotonin in the Tph2 (tryptophan hydroxylase 2, the rate-limiting enzyme for serotonin synthesis) knockout mice did not change the number of dopaminergic neurons (Gutknecht et al., 2012). However, recent optogenetic studies also showed interactions between the dopamine and serotonin systems, involving the mesolimbic system at the level of the ventral tegmental area in controlling motivation (McDevitt et al., 2014; Browne et al., 2019). Moreover, optogenetic stimulation of serotonergic terminals induced dopamine release from serotonin terminals following treatment with L-DOPA, with a loss of serotonin-mediated synaptic transmission (Gantz et al., 2015).

A depletion of striatal serotonin after dopaminergic lesions and in parkinsonian brains has been observed (Karstaedt et al., 1994; Rylander et al., 2010). However, in the neonatal brain, dopaminergic lesions with 6-hydroxydopamine (6-OHDA) led to striatal serotonergic hyperinnervation (Stachowiak et al., 1984; Snyder et al., 1986; Towle et al., 1989; Avale et al., 2004; Brown and Gerfen, 2006). In adult rodents, the sprouting of striatal serotonergic afferents was observed after dopaminergic lesions (Zhou et al., 1991; Guerra et al., 1997; Rozas et al., 1998; Maeda et al., 2003). Interestingly, dopamine-rich intrastriatal grafts did not prevent or revert the 6OHDA-induced serotonergic hyperinnervation (Guerra et al., 1997). Consistent with hyperinnervation, 6-OHDA lesions significantly increased firing discharges of serotonin neurons (Zhang et al., 2007; Kaya et al., 2008; Wang et al., 2009; Prinz et al., 2013). However, other studies did not observe changes (Miguelez et al., 2011, 2016) or even a decrease in the firing activity (Guiard et al., 2008). An increase in serotonin levels only in the first week (Silva et al., 2016) and a decrease of SERT availability after the 6-OHDA lesion (Walker et al., 2019) was also observed. Different experimental protocols, age of the animals, site of injection, and survival time after lesion may explain discrepancies between studies.

Interactions between dopaminergic and serotonergic systems were also observed during development (Lauder, 1990). In rat mesencephalic precursors, the reduction of serotonin levels induced an increase in the differentiation of dopaminergic neurons. Conversely, serotonin decreased the generation of dopaminergic neurons from mesencephalic precursors *via* serotonin type 7 and type 4 receptors (Rodriguez-Pallares et al., 2003; Parga et al., 2007).

### Interactions With the Glutamatergic System

In the basal ganglia, several studies have shown interactions of dopaminergic and serotonergic afferents with corticostriatal glutamatergic terminals. Fenfluramine is a halogenated amphetamine derivative thought to induce serotonin release and to reduce re-uptake. Fenfluramine induced striatal expression of Fos (used as a neuronal activity marker), which was reduced by dopaminergic and serotonergic lesions and suppressed by NMDA glutamate receptor antagonists, suggesting that stimulation of glutamate receptors is essential for the observed neuronal response (Guerra et al., 1998). Furthermore, Fenfluramine induced an increase in striatal levels of preproenkephalin mRNA, and this increase was blocked by dopamine receptor antagonists, NMDA glutamate receptor antagonists, or serotonergic lesions (Liste et al., 2000). These interactions are also supported by other studies showing that intraneuronal signaling pathways may interact to regulate gene transcription in the striatum (Ciranna, 2006). Consistent with this, 5-HT1A activation decreased glutamate release from corticostriatal projections (Dupre et al., 2013; Miguelez et al., 2014), and serotonergic denervation led to the loss of the serotonin inhibitory control on glutamate release (Vermeiren et al., 2018). It was also observed that a glutamatergic projection arising from the DRN-VGluT3 neurons provide excitatory synaptic input to the mesoaccumbens dopamine neurons. The discovery of this pathway opens new avenues to examine its participation in mental disorders related to motivation (Qi et al., 2014). Furthermore, a path-specific input from DRN serotonergic neurons to the ventral tegmental area promotes reward by the release of glutamate and activation of mesoaccumbens dopamine neurons (Wang H. L. et al., 2019). Interestingly, DRN serotonin neurons receive both excitatory and inhibitory inputs from the same brain areas, including the susbstantia nigra and cerebral cortex, to control neuronal activity (Zhou et al., 2017).

### Interactions With the Noradrenergic System

Although it is usually considered that the striatum is not significantly innervated by the noradrenergic system, locus coeruleus neurons send direct projections to the main striatal afferent systems, including the serotonergic system (Aston-Jones and Grzanna, 1995). The lack of serotonin innervation in the Tph knockout mice model induces a reduction in the number of noradrenergic neurons and noradrenaline levels in the locus coeruleus (Gutknecht et al., 2012; Pratelli and Pasqualetti, 2019). Analysis of the striatal responses to amphetamine is a useful tool to study the interaction between the noradrenergic and serotonergic systems. Amphetamine acts by increasing dopamine levels, but other neurochemical systems are also involved. The α1-adrenergic receptor antagonist Prazosin or lesions of the serotonergic system reduced locomotor activity and the striatal expression of Fos induced by amphetamine (Muñoz et al., 2003). These results showed that the noradrenergic and serotonergic systems play an important role in modulating the activity of striatal neurons. Other studies were consistent with this as they showed that the release of serotonin is subjected to noradrenergic influence mediated by α1-adrenergic receptors and that administration of Prazosin reduces serotonin levels (Rouquier et al., 1994; Hjorth et al., 1995; Rea et al., 2010). Moreover, studies using the 6-OHDA model revealed that both noradrenaline and serotonin depletion contribute to dysregulation of the basal ganglia in PD (Delaville et al., 2012). Furthermore, both noradrenergic and serotonergic systems modulate neurotransmission in the prefrontal cortex, which is altered in several psychiatric and neurological disorders (Hensler et al., 2013). Indeed, the most widely used antidepressants are SSRIs and noradrenaline reuptake inhibitors. A electrophysiological study showed that L-DOPA did not modify the basal neuronal activity in the locus coeruleus, however, it enhanced the response to noradrenaline reuptake inhibitors and decreased the effect of SSRI antidepressants (Miguelez et al., 2013).

### Interactions With the GABAergic System

Serotonin exerts a modulatory action on the effects of gamma-amino-butyric acid (GABA), which is the main brain neurotransmitter mediating inhibitory signals. Deficiency in brain serotonin results in alterations in the GABAergic system (Pratelli and Pasqualetti, 2019). The use of low doses of diazepam is enough to induce effects in Tph2 −/− mice, while they are not effective in wild type mice (Mosienko et al., 2015). At the presynaptic level, serotonin inhibits GABA release *via* 5-HT_1A_ and 5-HT_1B_ receptors and stimulates GABA release *via* 5-HT_3_ and 5HT_2_ receptors. GABA-mediated effects can also be modulated by serotonin at a post-synaptic level through different receptors and mechanisms, as observed in pyramidal neurons from the prefrontal cortex, hippocampus or thalamus (Ciranna, 2006; Miguelez et al., 2014). In GABAergic neurons, selective 5-HT_1A_ receptor-mediated signaling paradoxically increases c-fos expression and induces excitation in the prefrontal cortex pyramidal neurons (Masana et al., 2012; Hensler et al., 2013). However, the interactions are complex because serotonin also modulates other neurotransmitters. A deeper knowledge of neurotransmitter interactions will provide useful strategies for the therapy of several diseases (see Table 1).

**Table 1:** Summary reporting the major findings obtained in the different topics.

TOPIC	Authors	Major Findings
Interaction with DA	Morelli et al. (2011)	SERT blockade using SSRIs reduces dopaminergic signaling leading basal ganglia disfunction.
	McDevitt et al. (2014) and Browne et al. (2019)	Optogenetic studies showed interactions between the dopamine and serotonin for controlling motivation.
	Guerra et al. (1997) and Rozas et al. (1998)	Dopaminergic lesions induced serotonergic hyperinnervation.
	Karstaedt et al. (1994) and Walker et al. (2019)	Dopaminergic lesions induced depletion of striatal serotonin.
	Parga et al. (2007)	Serotonin decreases the generation of dopaminergic neurons from mesencephalic precursors.
Interaction with GLU	Guerra et al. (1998) and Liste et al. (2000)	Fenfluramine-induced expression of Fos and preproenkephalin mRNA is suppressed by NMDA antagonists.
	Vermeiren et al. (2018)	Serotonergic denervation led to a loss of the serotonin inhibitory control on glutamate release.
	Wang H. L. et al. (2019)	DRN neurons projecting to ventral tegmental area promotes reward by the release of glutamate.
Interaction with NA	Gutknecht et al. (2012)	The knockout mice model induces a reduction in the number of noradrenergic neurons in locus coeruleus.
	Muñoz et al. (2003)	α1-adrenergic receptor antagonists reduced striatal expression of Fos induced by amphetamine.
	Miguelez et al. (2013)	L-DOPA decreased the effect of SSRI antidepressants in the locus coeruleus.
Interaction with GABA	Pratelli and Pasqualetti (2019)	Deficiency in brain serotonin using Tph2 −/− mice results in alterations of the GABAergic system.
	Ciranna (2006) and Miguelez et al. (2014)	GABA-mediated effects are modulated by serotonin in the cortex, hippocampus, and thalamus.
	Masana et al. (2012)	5-HT_1A_ receptor-mediated signaling increases c-fos expression in the cortical GABAergic neurons.
5HT in LID	Lopez et al. (2001)	The effects of exogenous L-DOPA were blocked when the serotonergic innervation was removed.
	Carta et al. (2007) and Muñoz et al. (2008)	Removal of serotonin afferents or dampening of serotonin activity by 5-HT_1A_ and 5-HT_1B_ agonists blocked LID.
	Rylander et al. (2010)	Dyskinetic monkeys and patients showed sprouting of serotonin terminals and increase in SERT levels.
	Ghiglieri et al. (2016)	Eltoprazine (a dual 5HT_1A/1B_ agonist) reduces LIDs by the regulation of synaptic plasticity.
	Kwan et al. (2020)	Compounds acting through 5-HT3 receptors reduced LID without impairing L-DOPA anti-parkinsonian action.
5HT in GID	Carlsson et al. (2009)	The inclusion of serotonergic neurons in the grafts exacerbated the development of GID.
	Politis et al. (2011)	The serotonin 5-HT_1A_ receptor agonist buspirone produced significant dampening of GID in grafted patients.

*Abbreviations: 5-HT, serotonin; DA, dopaminergic; GLU, glutamatergic; NA, noradrenergic; GABA, GABAergic system; PD, Parkinson disease; LID, L-DOPA induced dyskinesia; GID, graft-induced dyskinesia*.

## Serotonin and Parkinson’s Disease

PD patients and PD animal models showed serotonergic neuronal loss and Lewy bodies within serotonergic neurons (Paulus and Jellinger, 1991; Huot and Fox, 2013). Moreover, serotonin levels and SERT expression are reduced in several nuclei in PD (Ciranna, 2006; Rylander et al., 2010). However, several findings indicate that the loss of SERT is not correlated with the disease duration and disability (Politis et al., 2010a; Politis and Loane, 2011). In the basal ganglia, changes in receptor expression were also observed, such as the increase in 5-HT_2C_ levels and a decrease in 5-HT_1A_ expression (Fox and Brotchie, 2000; Ballanger et al., 2012). Nevertheless, the differential expression of these receptors between regions and discrepancies between different studies using PD models have also been published (Miguelez et al., 2014). Serotonin signaling modulates the RhoA/Rho kinase pathway (Mair et al., 2008; Tanaka et al., 2014), which is involved in neuroinflammation and neurodegenerative disorders such as PD (Labandeira-Garcia et al., 2015; Koch et al., 2018). In PD, serotonin dysfunction, together with the noradrenergic dysfunction (Vermeiren and De Deyn, 2017), are involved in non-motor symptoms such as depression, weight loss, fatigue, and sleep disturbances. Recent studies have shown that administration of the serotonin precursor 5-hydroxytryptophan improves depressive symptoms in PD patients (Meloni et al., 2020). Furthermore, serotonin dysregulation leads to motor alterations such as tremor, L-DOPA-induced dyskinesia (LID), and graft-induced dyskinesias (GID).

### Involvement of Serotonin in L-DOPA-Induced Dyskinesias

Evidence from animal and human studies shows that striatal serotonergic terminals may contribute to the development of LID by promoting a non- physiological release of dopamine (Carta et al., 2007; Rylander et al., 2010; Navailles and De Deurwaerdere, 2012; Politis et al., 2014; Jenner, 2018).

The efficacy of L-DOPA is attributed to its conversion into dopamine by the enzyme aromatic L-amino acid decarboxylase (AADC) in striatal dopaminergic terminals. However, in advanced stages of the disease, the dopaminergic denervation is almost complete and other cell types showing AADC activity convert exogenous L-DOPA into dopamine, including serotonergic terminals (Arai et al., 1994; Maeda et al., 2005), endothelial cells (Melamed et al., 1980), glial cells (Li et al., 1992), and monoaminergic or nonaminergic striatal neurons (Mura et al., 1995; Lopez-Real et al., 2003). It was initially suggested that L-DOPA may produce dopamine-like responses in the absence of dopamine release. Using the AADC inhibitor NSD-1015, we showed that rotation and striatal Fos expression induced by L-DOPA were absent (Lopez et al., 2001), indicating that these effects are not due to a direct action of L-DOPA and are due to its conversion to dopamine. In the same study, we found that the effects of exogenous L-DOPA were blocked by removing serotonergic innervation (Lopez et al., 2001). Interestingly, removal of serotonin afferents or dampening of serotonin activity by 5-HT_1A_ and 5-HT_1B_ agonists blocked LID in rat and primate models (Carta et al., 2007; Muñoz et al., 2008; Fisher et al., 2020). Serotonergic neurons can convert L-DOPA into dopamine, which is stored and released as a “false neurotransmitter.” However, serotonergic terminals are unable to regulate dopamine release due to the lack of regulatory feedback mediated by the dopamine transporter and type-2 dopamine autoreceptors. In this scenario, activation of serotonin autoreceptors by selective agonists reduces dopamine release dampening synaptic dopamine peaks and LID (Carta et al., 2008, 2010). Administration of higher doses of 5-HT_1A_ and 5-HT_1B_ agonists also suppressed apomorphine induced-dyskinesia but by a different mechanism involving the activation of postsynaptic 5HT_1_-receptors expressed in non-serotonergic neurons in different brain areas (Muñoz et al., 2009). Other studies provided further support about the key role of the serotonin in LID. Recent studies showed a selective regulation of 5-HT_1B_ serotonin receptor mRNA expression by L-DOPA treatment (Padovan-Neto et al., 2020), and dyskinetic monkeys and patients showed sprouting of serotonin terminals and increase in SERT levels (Rylander et al., 2010; Beaudoin-Gobert et al., 2018; Walker et al., 2019). BDNF overexpression increased the susceptibility to LID due to serotonin hyperinnervation (Tronci et al., 2017), and other recent studies further supported the role of BDNF in LID (Sanna et al., 2020). In addition to DA, other metabolic products released by the serotonin neurons such as trace amines, may also be involved in L-DOPA effects acting as “false neurotransmitters” (Chagraoui et al., 2019). The interaction between the serotonin system and L-DOPA is thought to be more relevant at terminal level, rather than at the somatic level, because no changes in serotonin neuron somas or serotonin levels were observed in the DRN of dyskinetic rats (Rylander et al., 2010; Bishop et al., 2012).

Compounds acting through the serotonin system such as anpirtoline, (Bézard et al., 2013) or eltoprazine (Ghiglieri et al., 2016), which are a dual 1A/1B affinity 5HT agonist, or 5-HT_2A_ antagonists (Meco et al., 2003; Frouni et al., 2019; Kwan et al., 2019) showed beneficial effects against LID. LIDs are accompanied by impairment in corticostriatal bidirectional synaptic plasticity (Picconi et al., 2003), and eltoprazine reduces LIDs by the regulation of long-term potentiation and synaptic depotentiation in striatal neurons (Ghiglieri et al., 2016). The role of SERT is also being explored as a possible target against LID, and SERT blockade with SSRIs is also effective. However, data from non- human primates treated with some of these drugs also led to worsening of parkinsonian symptoms. However, opposite results were also observed (Bishop et al., 2012; Conti et al., 2014; Fidalgo et al., 2015; Lanza and Bishop, 2018). Recently, Vilazodone, a selective SSRI, and a partial 5-HT_1A_ agonist have been shown to reduce LID without compromising L-DOPA efficacy (Meadows et al., 2018).

Clinical trials with serotonergic drugs are ongoing, revealing the promising antidyskinetic effects of 5HT_1A_ agonists such as buspirone (Politis et al., 2014), sarizotan (Bara-Jimenez et al., 2005; Goetz et al., 2007), and tandospirone (Kannari et al., 2002). However, these drugs, at high doses, may interfere therapeutic effects of L-DOPA, due to the presence of the autoreceptors in non-serotonergic neurons and possible antagonistic action on dopaminergic receptors. Eltoprazine could provide effective suppression of LID and a wider therapeutic window (Svenningsson et al., 2015; Frouni et al., 2019; Wang Q. et al., 2019). Recently, compounds acting through 5-HT_3_ receptors also reduced LID (Kwan et al., 2020). However, the pathophysiology of dyskinesia is complex as the glutamatergic system is also involved. An interesting possibility is to combine 5HT_1_ agonists with drugs that modulate the glutamatergic function (Tison et al., 2013; Carta and Björklund, 2018). Neuroinflammation and angiogenesis are also involved in the development of dyskinesia and are also therapeutic targets (Muñoz et al., 2014; Bishop, 2019; Boi et al., 2019).

### Involvement of Serotonin in Graft-Induced Dyskinesias

Clinical trials using transplants of fetal dopamine neuroblasts have shown promising results, although many patients have developed GID (Freed et al., 2001; Olanow et al., 2009; Bjorklund and Kordower, 2013; Li et al., 2016; Barker, 2019). The mechanism underlying GID is still unclear (Freed et al., 2001; Hagell et al., 2002; Barker and Kuan, 2010). Serotonergic neurons usually present in the grafted cell suspension contribute to serotonergic innervation of the ventral mesencephalic grafts and the surrounding striatum (Guerra et al., 1997). It has been suggested that the inclusion of serotonergic neurons in the grafted ventral midbrain tissue may lead to the development of GID (Politis and Loane, 2011; Shin et al., 2012b). Experimental studies using different proportions of dopamine and serotonin neurons in the grafted cell suspension showed that the increase in the number of serotonin neurons within the transplant led to progressive worsening of dyskinesia, and the relative density of dopamine and serotonin innervation in the grafted striatum appears as a critical factor, even more than the absolute number of serotonin neurons within the grafts (Carlsson et al., 2007, 2009). In PD patients with GID, grafted tissue contained a large number of serotonergic neurons and excessive graft-derived serotonergic innervation (Politis et al., 2011; Tronci et al., 2015). Moreover, the serotonin 5-HT_1A_ receptor agonist buspirone produced significant dampening of GID in grafted patients. However, this effect could also be explained by the dopamine D2 receptor partial antagonistic effects of the drug (Politis et al., 2010b, 2011; Shin et al., 2012a), and the long term effect of this compound is uncertain (Beaulieu-Boire and Fasano, 2015). Removal of the endogenous serotonin innervation abolished the anti-GID properties of the 5-HT_1A_ and 5-HT_1B_ agonists, suggesting that the effect of these drugs on GID is mediated by the activation of presynaptic host-derived receptors (Shin et al., 2012a). Nevertheless, dopamine receptor blockade in fetal mesencephalic grafts induces a striking enhancement of the antidyskinetic effect suggesting that both serotonergic and dopaminergic mechanisms may interact in the development of GID (Shin et al., 2014). Controversial data have shown high striatal 5-HT transporter content in the absence of graft-induced dyskinesia (Mendez et al., 2005; Lane, 2019). Studies using a new experimental model, in which the activity of the transplanted dopaminergic neurons can be selectively modulated using a bimodal chemogenetic approach (DREADD), revealed a novel dyskinesia mechanism mediated by the serotonin 5-HT_6_ receptors (Aldrin-Kirk et al., 2016). The next step should be to evaluate the impact of L-DOPA therapy on grafts from new cell sources, particularly human embryonic and induced pluripotential stem cells that will be used in upcoming clinical trials (Kirkeby et al., 2017; Studer, 2017; Table 1).

## Concluding Remarks

Interactions between serotonergic and other neurotransmitter systems reveal that serotonin plays a crucial role in the control of movement by the basal ganglia. These interactions are of great interest for understanding the pathophysiology of PD and to develop novel therapeutic strategies. Manipulation of the serotonergic system represents a valuable target to treat LID and GID in PD patients. However, further investigation is required to clarify mechanisms of neurotransmitter interactions and to determine optimal compounds and doses for effective therapies.

## Author Contributions

All authors have contributed to this work and approved its final version for submission. AM developed the idea for this review and wrote the manuscript. AL-L, CL, and JL-G prepared the figure and were involved in the literature review and preparation and revision of the manuscript.

## Conflict of Interest

The authors declare that the research was conducted in the absence of any commercial or financial relationships that could be construed as a potential conflict of interest.
